# The Memorial to Sir Alfred Jones

**Published:** 1913-07-12

**Authors:** 


### THE MEMORIAL TO SIR ALFRED JONES.

On Saturday last the citizens of Liverpool assembled on the Pier Head to witness the unveiling of a statue, for which they had subscribed, of the late Sir Alfred Jones, one of the greatest merchant princes that England has owned. As all the world knows, the commercial enterprises initiated and controlled by this distinguished man were of colossal magnitude and of world-wide character. They were characterised also by a practical imperialism and a far-seeing faith in the future of our Colonies which achieved results as remarkable as those of many of our empire-builders themselves. And of all the consequences which sprang from his many-sided activity and power of organisation there is none which has surpassed (or is likely to outlast) the result of his appreciation of the hygienic problems of residence in the tropics. Those problems are by no means yet solved; but we are justified in saying that the last twenty years have seen more progress than the previous twenty centuries, and that science marches steadily from victory to victory in the battle with tropical diseases. Presently we shall have the completed Panama Canal as a material witness to the conquest of the tropics; and there are other enterprises which scientific research alone has rendered feasible. It is not too much to say that the development of the tropics during this twentieth century is going to be one of the really big and vital factors in political economy and in *Welt-politik*; and that development rests on a foundation laid in the last two decades chiefly by Englishmen, many of whom were equipped by the Liverpool School of Tropical Medicine and its financial backer, Sir Alfred Jones.

In commemorating Alfred Jones, the trader, the shipowner, the banker, the developer of West Africa and the West Indies, Liverpool honours one who deserved well of his city. But as the organiser and the ever-liberal endower of tropical research, he deserved better still of the State and of humanity at large. It is true that he himself did not adventure into the forefront of the battle; and that the heroism of the members of his expeditions won them renown more glorious than his. Yet it is not the privilege of the modern generalissimo to risk his life in action as of old; duty demands that he shall occupy a safe position miles in rear of the firing line. So did Sir Alfred Jones direct the energies and supply the needs of his soldiers; and in all their victories for civilisation and for humanity he takes a rightful share. Liverpool and Lancashire have done well thus promptly to show that "some love England and her honour yet," which the poet declared sixty years ago that one important section of that county did not. In days to come, when men look back at the works of the tropical hygienists of the last two decades, and trace out their astounding consequences, a roll of honour will be prepared upon which many names will be inscribed; most of them, we are proud and glad to say, our countrymen: many of them still living. When that time comes the share taken by Sir Alfred Jones in promoting these enterprises will assuredly not be forgotten.

